# Quantifying physical insights cooperatively with exhaustive search for Bayesian spectroscopy of X-ray photoelectron spectra

**DOI:** 10.1038/s41598-023-40208-3

**Published:** 2023-08-14

**Authors:** Hiroyuki Kumazoe, Kazunori Iwamitsu, Masaki Imamura, Kazutoshi Takahashi, Yoh-ichi Mototake, Masato Okada, Ichiro Akai

**Affiliations:** 1 Graduate School of Social Data Science, Hitotsubashi University, Kunitachi, Tokyo 186-8601 Japan; 2 Technical Division, Kumamoto University, Kumamoto, 860-8555 Japan; 3 Synchrotron Light Application Center, Saga University, Tosu, Saga 841-0005 Japan; 4 Department of Complexity Science and Engineering, The University of Tokyo, Kashiwa Chiba, 277-8561 Japan; 5 Research and Services Division of Materials Data and Integrated System, National Institute for Materials Science, Tsukuba, Ibaraki 305-0047 Japan; 6 Institute of Industrial Nanomaterials, Kumamoto University, Kumamoto, 860-8555 Japan

**Keywords:** Optics and photonics, X-rays, Theory and computation

## Abstract

We analyzed the X-ray photoelectron spectra (XPS) of carbon 1s states in graphene and oxygen-intercalated graphene grown on SiC(0001) using Bayesian spectroscopy. To realize highly accurate spectral decomposition of the XPS spectra, we proposed a framework for discovering physical constraints from the absence of prior quantified physical knowledge, in which we designed the prior probabilities based on the found constraints and the physically required conditions. This suppresses the exchange of peak components during replica exchange Monte Carlo iterations and makes possible to decompose XPS in the case where a reliable structure model or a presumable number of components is not known. As a result, we have successfully decomposed XPS of one monolayer (1ML), two monolayers (2ML), and quasi-freestanding 2ML (qfs-2ML) graphene samples deposited on SiC substrates with the meV order precision of the binding energy, in which the posterior probability distributions of the binding energies were obtained distinguishably between the different components of buffer layer even though they are observed as hump and shoulder structures because of their overlapping.

## Introduction

X-ray core-level photoelectron spectroscopy (XPS) is a popular and powerful tool for investigating the elemental composition of materials^1,2^. Especially, due to the short escape depth of photoelectrons excited by soft X-rays, XPS has been applied to various surface and interface analysis such as film thickness^3^, chemical states at surfaces or interfaces^4–6^, and atomic distortion at the interface^7^. The elemental information measured by XPS is revealed by line-shape analysis for the obtained spectrum. Although the regression analysis of XPS spectra is a non-linear regression, the least squares method has been used to minimize the fitting error until now. In an analysis using the least-squares method, it is not always possible to obtain a globally optimal solution, and its reproducibility is frequently low because it depends on the initial values. It is also difficult to incorporate known physical property information such as constraints on the parameters, and it is impossible to evaluate the accuracy of the parameter estimation. In the analysis of XPS, it is essential to develop a highly reproducible analysis method that properly incorporates known physical property information^8,9^. In this paper, to solve these problems, we apply Bayesian spectroscopy to analyze the XPS spectra of one monolayer (1ML), two monolayers (2ML), and quasi-freestanding 2ML (qfs-2ML) graphene layers grown on SiC substrates and attempt to extract changes in the chemical states of the graphene layers by chemical modification at the interface.

High-quality, large-scale graphene can be formed by thermal decomposition of SiC at elevated temperatures. It is known that buffer layer are formed between graphene and the SiC substrate and have an equivalent structure to graphene, although the buffer layer adhere strongly to the SiC substrate, while dangling bonds remain with the SiC substrate due to lattice mismatch^5^. Riedl et al. proposed that graphene C 1s spectra have four peaks, in addition to SiC and graphene (Gr), two additional components S1 and S2^5^. S1 comes from the C atoms bound to one Si atom on the surface of SiC(0001) and to three C atoms in the buffer layer. S2 comes from the remaining sp$^2$-bonded C atoms in the buffer layer. The carbon layer on this buffer layer exhibits the properties of graphene. To modify the bonding at the interface, various atoms, such as H^10^, O^11,12^, Ge^13^, Si^14^, Au^15^ and Bi^16^ have been intercalated between the buffer layer and SiC. When atoms are intercalated beneath the buffer layer, dangling bonds of Si are terminated by intercalated atoms to break covalent bonds between the buffer layer and SiC. Since the charge transfer into the graphene layer is also modified by the intercalation, the charge neutrality level can be controlled around the Dirac point artificially. However, the transport properties are often degraded after the interface modification. To enhance the transport property of graphene, precise control and characterization of the chemical states should be performed at the intercalated interface. The bonding at the modified interface would differ depending on the intercalated atoms and on the treatment conditions.

In such cases, the reliable structure model for the intercalated interface would often be lacking. Thus, an alternative method is strongly required to decompose core-level photoelectron spectra even in the case where a reliable structure model or a presumable number of components is not known. However, we have been forced to analyze the lineshape with the number of components and the constraint parameters assumed to validate a plausible structure and the experimental setting. Thus, there might be a concern of containing subjective and empirical arbitrariness in the fitting results in the XPS spectra. In addition, if the line-shape analysis was treated as a black-box tool due to its complexity, that would yield incorrect results. Therefore, a reproducible and reliable approach without arbitrariness is required for XPS spectral analysis.

Recently, the result of Bayesian spectral deconvolution for core-level XPS has been reported to realize automatic analysis of core-level XPS spectra by incorporating the effective Hamiltonian into the stochastic model of spectral deconvolution^17^. 3d core-level XPS spectra of $\text {La}_2\text {O}_3$ and $\text {CeO}_2$ were well reproduced and it was confirmed that the effective Hamiltonians selected by model selection were in good agreement with the results obtained from a conventional study. The uncertainty of its estimated values, which are difficult to obtain with the conventional analysis method, and the reason why the effective Hamiltonian selected were also revealed by spectral deconvolution based on Bayesian inference^17^. In Japan, efforts to realize spectral decomposition based on Bayesian inference have begun in synchrotron radiation facilities^18^.

The difficulty in performing such Bayesian inference is in designing the prior probability distribution. Since prior probability distributions can restrict the range of parameters, if the constrain condition for their parameters is known in advance, it can be incorporated into the prior probability distribution. Otherwise, a distribution that does not affect parameter estimation, such as a wide uniform distribution, is used as the prior probability distribution. In that case, the posterior probability distribution may exhibit multimodality due to exchange among spectral components. As a result, parameter estimates are not precise, are poorly reproducible, and may not be physically reasonable. When analyzing the XPS spectra, it is important to overcome these difficulties and quantify knowledge of the target material^9^. However, even in such a situation, scientists can achieve high-precision spectral analysis by the following scenario. They first analyze the spectral data without assuming prior knowledge. By reviewing the results of this analysis, they quantify physical constraints that were previously unnoticed or unquantifiable. Utilizing such knowledge, for example, by constraining the range of regression parameters, they can achieve the analysis of spectral data with the desired accuracy. The purpose of this study is to propose a framework for incorporating this natural flow of spectral data analysis conducted by scientists into Bayesian spectroscopy^19,20^.

In this article, core-level spectra in both pristine and oxygen-intercalated graphenes grown in SiC(0001) have been analyzed by Bayesian spectroscopy with constraints on the values of the spectral parameters based on knowledge of the physical properties.

## Samples

Graphene layers were grown on *n*-doped 6H-SiC(0001) using the face-to-face method^21^, where two SiC substrates were placed one on top of the other with a gap of 20 $\upmu$m using Ta foils. After sufficient outgassing at approximately 800 $^\circ$C and annealing at 1200 $^\circ$C to provide a well-ordered Si-terminated surface, samples were annealed at 1350 $^\circ$C and 1400 $^\circ$C to obtain 1ML and 2ML graphene, respectively. Qfs-2ML were obtained by annealing the 1ML sample for 10 min at 550 $^\circ$C in air. All measurements were performed on the beamline BL13 at the SAGA Light Source^22^. The core-level and valence-band photoelectron spectra were measured using a photon energy of 680 and 40 eV, respectively. The Fermi energy and the energy resolution were confirmed by measurements for the Fermi level of the gold reference. The overall energy resolutions were estimated to be 0.69 and 0.04 eV for core-level and valence band measurements, respectively.

**Figure 1:**
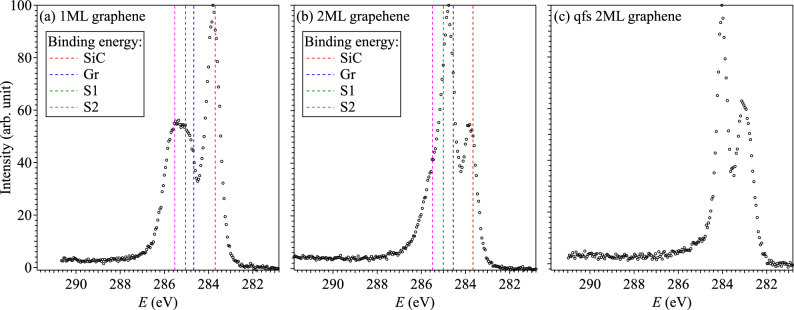
XPS spectra measured at C 1s level in 1ML (**a**), 2ML (**b**), and qfs-2ML (**c**) graphenes, respectively. Vertical dashed lines in (**a**) and (**b**) show the binding energies of SiC, graphene, S1, and S2 for 1ML and 2ML graphene reported in Ref.^23^.

Figure 1 shows the XPS spectra measured for 1ML, 2ML, and qfs-2ML graphene samples. Vertical dashed lines indicate the energy positions reported in a previous work^23^ for the 1ML and 2ML samples, which are shown in red for the SiC substrate, blue for graphene (Gr), green for S1 and magenta for S2, respectively. Although the peak positions are shifted about 0.2 eV comparing the vertical dashed lines with the peak positions observed in Fig. 1, this energy shift is considered to be due to differences in measurement conditions such as temperature and SiC doping concentration because the binding energy scale was calibrated with respect to the Fermi level of the gold reference.

In the 1ML sample depicted in Fig. 1a, the SiC substrate (283.70 eV^23^) gives the strongest peak at 283.8 eV, and the peak structures of the graphene (284.67 eV^23^) and of the two components S1 (285.04 eV^23^) and S2 (285.53 eV^23^) for buffer layer atoms are observed as broad peaks from 284.5 to 286.0 eV without separation. In the 2ML sample shown in Fig. 1b, the SiC substrate (283.66 eV^23^) gives a second intense peak at 283.3 eV, which is the almost same as that of 1ML. However, the spectral structure in the high-binding energy region changes markedly, giving a dominant peak at 284.8 eV. Although this peak is considered to be chiefly attributed to graphene (284.56 eV^23^), and also includes the components of the buffer layer S1 (285.01 eV^23^) and S2 (285.50 eV^23^) because it has a shoulder structure on the high energy side.

The surface cleanliness and the thickness of graphene were checked by the core-level and valence-band photoemission measurements. No contamination-related features such as an O 1s peak were found in the core-level photoemission spectra. Angle-resolved photoemission spectra (ARPES) showed clear dispersions originating from the $\sigma$ and $\pi$ bands along the $G-K$ direction. In particular, the thickness dependent dispersions of 1ML and 2ML graphene were observed around the ${\overline{K}}$ point, consistent with the previous report^24^.

The buffer layer at the interface between SiC and graphenes consists of a carbon layer in a graphene-like honeycomb arrangement that bonds covalently to the Si-terminated substrate partially. For the 1ML and 2ML samples, not all Si atoms can bond to carbon atoms due to the different lattice constants between SiC and graphene and due to the $30^{\circ }$ rotation angle of the carbon layer relative to the SiC substrate. Although the covalent bond breaks the hexagonal network of $\pi$ orbitals but preserves the $\sigma$-bonds^5,25,26^, it is not known whether the Si dangling bonds remain in the sample qfs-2ML after annealing. The XPS spectrum in the qfs-2ML sample is similar to that in the 2ML sample, as seen in Fig. 1, in which they have two main peaks and a shoulder in the higher energy peak. Thus, it is considered that the two main peaks at 283.1 and 284.0 eV come from the SiC substrate and graphene. However, in the qfs-2ML sample, it is controversial that there are both buffer layer components (S1 and S2) on the shoulder of the 284.0 eV peak. Furthermore, one can find that the peak positions for the qfs-2ML sample are shifted to the lower binding energy side by about 0.7 eV in Fig. 1, although the energy axis was calibrated, and the physical reason for this shift remains elusive. Although we need to discuss the bonding properties of carbon from multiple aspects, not only the C 1s level but also the Si 2p and/or O 1s levels, we focus on the C 1s level in this paper. This is because the characterization of the graphene has already been reported^24^ and the objective is to confirm the validity of our proposed framework.

## Framework

**Figure 2:**
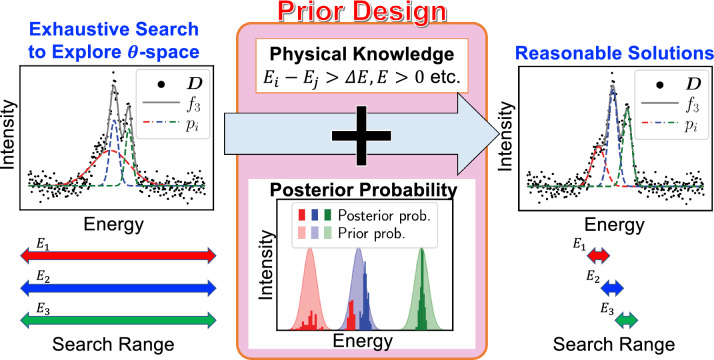
Schematic of our analysis procedure. First, we perform an exhaustive search to explore the parameter space. Next, we configure a prior probability distribution based on the constraints of physical properties and/or insights gained from the obtained posterior probability distribution. Finally, we perform the analysis again to estimate the parameters.

Figure 2 is the schematic diagram of our proposed framework. To realize the spectral decomposition with high precision, we performed the following two-step analysis. First, we performed an exhaustive search using uniform distributions for the prior probability of parameters $\varvec{\theta }_{K}$ on the respective spectral components of Gr, S1, S2, and SiC in order to explore $\varvec{\theta }$-space. We obtained posterior distributions of $\varvec{\theta }_{K}$ which exhibit that the actual $\varvec{\theta }$-space might be occupied by parameters. Here, we design the prior probability distribution on the basis of the posterior distributions obtained by the exhaustive search for the analysis in the next step. When prior information is available, we can incorporate it into prior probability distributions. Next, we analyzed the target data, $\varvec{D}$ using the designed prior probability distribution for estimating $\varvec{\theta }_K$. In Fig. 2, the XPS spectrum $\varvec{D}$ contains three peaks $p_i (i = 1, 2, 3)$^19,27^. However, applying uniform distribution of the prior probability of energy *E*, the width of the red component is large and the obtained solution is not reasonable as a result of XPS as shown in the left of Fig. 2, and the precise estimation of the binding energy *E* is inhibited because the posterior distribution of the red component obtained by the exhaustive search becomes two-modal and one distributed near the blue component as shown in Fig. 2. Here, we design the prior probability distribution of *E*. When we have constrain conditions for *E*: each energy difference is greater than $\Delta E (> 0)$, we can incorporate the physical property that the red component is not located around the blue component. Thus, we can configure prior probability as demonstrated in Fig. 2, and obtain reasonable solutions with high precision.

### Bayesian spectroscopy

Bayesian spectroscopy is a spectral decomposition analysis method that incorporates a Bayesian inference framework. Let $\varvec{D} = \{ (x_i, y_i) \mid i = 1, \cdots , N \}$ be a data set of an XPS spectrum and $f_{K}(x_i; \varvec{\theta }_{K})$ be a phenomenological model function to describe $\varvec{D}$, where *K* is a subscript for model identification. Based on $\varvec{D}$, Bayesian spectroscopy evaluates the posterior probability distributions of the material-specific parameters $\varvec{\theta }_{K}$ in the model function to be estimated.

From Bayes’ theorem^28,29^, the posterior probability distribution $P(\varvec{\theta }_{K}|\varvec{D},b,K)$ is given by Eq. (1).

1 $$\begin{aligned} P(\varvec{\theta }_{K}|\varvec{D},b,K) = \frac{ P(\varvec{D}|\varvec{\theta }_{K},b,K) P(\varvec{\theta }_{K}|b,K) }{ P(\varvec{D}|b,K) }, \end{aligned}$$

where *b* is the quasi-inverse temperature^27^ defined as an inverse variance $b = \sigma _{\text {noise}}^{-2}$ with a standard deviation $\sigma _{\text {noise}}$ of the superimposed noise in $\varvec{y}=\{y_i\mid {}i=1,\cdots ,N\}$. When the noises in $\varvec{y}$ are distributed independently in *i* according to a normal distribution with zero mean and variance $b^{-1}$, the likelihood term $P(\varvec{D}|\varvec{\theta }_{K},b,K)$ is given by $P(\varvec{D}|\varvec{\theta }_{K}, b) = \left( b/2\pi \right) ^{N/2} \exp \left[ - b N {\mathscr {E}}_{K}(\varvec{\theta }_{K}) \right]$ with an error function ${\mathscr {E}}_{K}(\varvec{\theta }_{K})$ defined in Eq. (2).

2 $$\begin{aligned} {\mathscr {E}}_{K}(\varvec{\theta }_{K}) = \frac{1}{2N} \sum _{i=1}^{N} \left[ y_i - f_{K}(x_i; \varvec{\theta }_{K}) \right] ^2. \end{aligned}$$

A Bayesian partition function *Z*(*b*, *K*)^19^ is obtained by marginalizing the numerator of Eq. (1) over $\varvec{\theta }_{K}$:

$$\begin{aligned} Z(b,K) \equiv P(\varvec{D}|b,K) = \left( \frac{b}{2\pi } \right) ^{N/2} \int \exp \left[ - b N {\mathscr {E}}_{K}(\varvec{\theta }_{K}) \right] P(\varvec{\theta }_{K}|b,K) \, d \varvec{\theta }_{K}, \end{aligned}$$

and a Bayesian free energy^19^ (BFE) is defined as $F(b,K)=-\ln {}Z(b,K)$. By minimizing *F*(*b*, *K*), the estimation of the noise intensity ${\hat{\sigma }}_\text {noise}$ superimposed on the measured data $\varvec{D}$ and the model selection of the most appropriate function $f_{\hat{K}}(x_i;\varvec{\theta }_{\hat{K}})$ to explain $\varvec{D}$ can be achieved simultaneously by Eq. (3).

3 $$\begin{aligned} \{\hat{b}, \hat{K}\} = \mathop {\mathrm {arg\,min}}\limits _{b, K} F(b, K), \,\, {\hat{\sigma }}_\text {noise} = \hat{b}^{-1/2}. \end{aligned}$$

The posterior probability distribution of material-specific parameters $\varvec{\theta }_{\hat{K}}$ is sampled using a replica exchange Monte Carlo (RXMC)^30^ method according to Eq. (4).

4 $$\begin{aligned} P(\varvec{\theta }_{\hat{K}}|\varvec{D},\hat{b},\hat{K}) \propto \exp \left[ - \hat{b} N {\mathscr {E}}_{\hat{K}}(\varvec{\theta }_{\hat{K}}) \right] P(\varvec{\theta }_{\hat{K}}|\hat{b},\hat{K}), \end{aligned}$$

and the maximum a posteriori probability (MAP) estimate in Eq. (5) is used for the optimal parameters $\hat{\varvec{\theta }}_{\hat{K}}$ to explain the measured data $\varvec{D}$.

5 $$\begin{aligned} \hat{\varvec{\theta }}_{\hat{K}} = \mathop {\mathrm {arg\,max}}\limits _{\varvec{\theta }_{\hat{K}}} P(\varvec{\theta }_{\hat{K}}|\varvec{D},\hat{b},\hat{K}). \end{aligned}$$

In spectral decomposition, when there is no prior knowledge of material-specific parameters, its prior probability $P(\varvec{\theta }_{K}|b,K)$ in Eq. (1) should not be restricted. However, we have to search the high-dimensional parameter space extensively, and rejections^31^ of candidate parameters prepared in Monte Carlo steps and exchange^31^ of spectral components during sampling become frequent, making sampling convergence difficult. On the other hand, when one wants to decompose spectra of specific materials and quantitatively evaluate changes in physical properties associated with changes in the material interface, as is the case in this paper, we can make positive efforts to incorporate the knowledge of material properties into the prior probabilities in Bayesian spectroscopy.

### Phenomenological model for the XPS spectrum

A phenomenological model $f_{K}(x_i;\varvec{\theta }_{K})$ in Eq. (6) is used for the spectral decomposition, which is a sum of peaks with a pseudo-Voigt function^32^ and the Shirley background signal^1^:

6 $$\begin{aligned} f_{K}(x_i; \varvec{\theta }_{K}) = \sum _{k=1}^{K} p(x_i;\varvec{\theta }_k^{\text {peak}}) + \frac{h}{C} \int _{-\infty }^{x_i} \sum _{k=1}^{K} p(\xi ;\varvec{\theta }_k^{\text {peak}}) \, d\xi , \end{aligned}$$

where *K* is the number of peaks in the XPS spectrum and $\varvec{\theta }_{K} = \{ \varvec{\theta }_{1}^{\text {peak}}, \cdots , \varvec{\theta }_{K}^{\text {peak}}, h \} .$ $p(x; \varvec{\theta }^{\text {peak}})$ is a pseudo-Voigt function in Eq. (7), which is a linear combination of Lorentzian *L*(*x*) and Gaussian *G*(*x*) shapes, with their intensity *A*, binding energy *E*, spectral width *w* at full width at half maximum (FWHM) and a mixing ratio $\eta$.

7a $$\begin{aligned} p(x; A, E, w, \eta )= & {} \eta \cdot L(x; A, E, w) + (1 - \eta ) \cdot G(x; A, E, w), \end{aligned}$$

7b $$\begin{aligned} L(x; A, E, w)= & {} A \frac{2}{\pi } \frac{w}{4(x - E)^2 + w^2}, \end{aligned}$$

7c $$\begin{aligned} G(x; A, E, w)= & {} A \sqrt{\frac{4 \ln 2}{\pi w^2}} \exp \left[ - 4 \ln 2 \left( \frac{x - E}{w} \right) ^2 \right] . \end{aligned}$$

*C* in Eq. (6) is a normalization constant for the Shirley background given by $\sum _{k=1}^{K} A_k$ and *h* is the height of the background signal in $x_i \rightarrow \infty$ where the intensity of the peaks must be zero and only the background signal remains.

Of course, other functions can be used. For example, the Doniach-Šunjić function^33^ convoluted with a Gaussian which gives asymmetric peaks and the Tougaard method^34^ which is more accurate for quantification than the Shirley method. We use the *simple* function, in order to confirm the effectiveness of the proposed method.

### Computational details

For RXMC sampling, we prepared 100 replicas with quasi-inverse temperatures $b_\ell$, $b_1 = 0$ and a geometric sequence $b_{\ell }$ for $2\le \ell \le {}100$ with $b_2=10^{-4}$ and $b_{100}=10$. In all analyses in this paper, the $\hat{b}$ obtained in Eq. (3) are 0.75 – 1.53, which falls within this $b_{\ell }$ range. On the other hand, $b_{2}$ should be chosen so that the state exchange of the set of parameters $\varvec{\theta }_{K}$ between the replica at $b_{1}$ ($=0$) is guaranteed. In this study, we set a sufficiently small $b_{2}$ and confirmed that the average exchange ratios between these replicas are more than 90 % in all analyses.

RXMC sampling was carried out in 1,000,000 steps after a sufficient burn-in phase of 600,000 steps. We used the auto-tuning algorithm^31^ for the step widths to achieve the mean acceptance ratio of 70 %.

## Incorporation of physical properties into prior probability

In addition to model selection^19^, the advantage of Bayesian spectroscopy is that appropriate knowledge of physical properties can be incorporated into the prior probability $P(\varvec{\theta }_{K}|b,K)$ in Eq. (1). In XPS, broad peaks sometimes arise from multiple components. Therefore, to perform a well-founded physical analysis with high precision, the restriction in the prior probability $P(\varvec{\theta }_{K}|b,K)$ for $\varvec{\theta }_{K}$ is especially effective.

**Table 1:** Prior probabilities of the binding energy $E_{k}$ expressed in eV. ^a^ ${\mathscr {N}}(\Delta {}E_1{\mathrm{ML}}, 0.07)$ is a prior probability for the energy difference $\Delta {}E$ ($=E_{{\mathrm{S}}2}-E_{{\mathrm{S}}1}$) of the binding energies between S2 and S1 components.

Components	1ML	2ML	Ref.^23^	qfs-2ML
SiC	${\mathscr {N}}(283.78, 0.022)$		283.70	${\mathscr {N}}(283.00, 0.040)$
Gr	${\mathscr {N}}(284.76, 0.026)$		284.67	${\mathscr {N}}(284.02, 0.0099)$
S1	${\mathscr {N}}(284.92, 0.33)$		285.04	${\mathscr {N}}(284.92, 0.20)$
S2	${\mathscr {N}}(285.61, 0.19)$	${\mathscr {N}}(\Delta {}E_{1{\mathrm{ML}}}, 0.07)$^a^	285.53	${\mathscr {N}}(284.92, 0.20)$

### Prior probability for binding energy

To associate each spectral component with each physical origin while suppressing component exchange during RXMC sampling, we set different prior probabilities for the binding energies $E_{k}$ to distinguish the respective components ($k=\mathrm {1:SiC, 2:Gr, 3:S1, 4:S2}$).

To accomplish this task by merging the results of the exhaustive search and the knowledge of the previous study, we divide the posterior probability distributions of $E_{k}$ obtained by the exhaustive search into four monomodal ones with reference to the previous study^23^ and evaluate the means $\mu _{k}$ and standard deviations $\sigma _{k}$ of the respective monomodal posterior probability distributions. For the prior probability of $E_{k}$, we use normal distributions ${\mathscr {N}}(E_{k};m_{k},s_{k})$ in Table 1, where $m_{k}$ and $s_{k}$ are their mean and standard deviation and are determined as $m_{k}=\mu _{k}$ and $s_{k}=5\sigma _{k}$. Although prior probabilities are used here with standard deviations larger than those evaluated in the exhaustive search, this setting avoids imposing excessive restrictions and allows the search for the parameter space of $E_{k}$ explored in the exhaustive search.

For 1ML and 2ML samples, we use the same prior probabilities in the respective components of SiC, Gr, and S1 as seen in Table 1 since the binding energies of these components are approximately equal in 1ML and 2ML samples, as confirmed in Figs. 1a,b. In the case of the 2ML sample in Fig. 1b, the S2 component appears as a shoulder structure associated with the strong components S1 and Gr. So, although there is a previous study^23^ showing that the binding energy of the S2 component does not differ significantly between the 1ML and 2ML samples, in the 2ML sample, the prior probability of the binding energy for the S2 component is designed as follows: we consider the difference $\Delta {}E$ ($\Delta {}E=E_{{\text {S}}2}-E_{{\text {S}}1}$) in the binding energy of the S2 component from the S1 component and introduce a prior probability of a normal distribution for $\Delta {}E$ as shown in Table 1, where the mean and standard deviation are $\Delta {}E_{1\mathrm{ML}}$ of the 1ML sample and 0.07 eV, respectively.

In the case of the qfs-2ML sample, although the entire XPS spectrum in Fig. 1c shifts to the lower energy side than those of the 1ML and 2ML samples, we can determine the prior probabilities of the binding energies for the SiC and Gr components as seen in Table 1 based on the exhaustive search in the same manner. However, the posterior probability distributions of the binding energies for S1 and S2 obtained in the exhaustive search are broad, and whether the S1 component remains in the qfs-2ML sample is controversial. Therefore, we prepare a prior probability of the normal distribution for S1 and S2 that has the same mean value as the S1 component in the 1ML and 2ML samples as shown in Table 1, and a large standard deviation of 0.20 eV is used to detect the energy shift of the components S1 and S2 in the qfs-2ML sample.

### Prior probabilities of other parameters

In the exhaustive search, the posterior distributions of the spectral width *w* are distributed in the range of less than about 4 eV and had a mode at about 0.9 eV. Thus, we use the same gamma distribution; ${\mathscr {G}}(x;\alpha ,\beta ) = \beta ^{\alpha } x^{\alpha - 1} e^{- \beta x} /\Gamma (\alpha )$ for the prior probability distribution of the nonnegative *w* in all components of all data, where $\Gamma (\alpha )$ is a gamma function, $\alpha =2.138$ and $\beta =1.265\,\mathrm{eV}^{-1}$, respectively. Therefore, the mode value of ${\mathscr {G}}(x;\alpha ,\beta )$ is 0.9 eV and the range of two standard deviations from the mean covers 4 eV. Although, to decompose the shoulder structure in the XPS spectrum of the 2ML sample [see Fig. 1b] into two components, the binding energy of S2 was parameterized by $\Delta {}E$ , we also assume that S1 and S2 have the same spectral width ($w_{{\mathrm{S}}1} = w_{{\mathrm{S}}2}$) in the 2ML sample, since these two buffer components are expected to have similar lineshapes. For other parameters in Eq. (6), we set $A>0$, $0\le \eta \le 1$, $h>0$ as prior probabilities using uniform distributions.

## Results

### 1ML and 2ML samples

**Table 2:** MAP estimates and standard deviations of $P(\theta |\varvec{D}, \hat{b})$ as measures of the precision of the estimation of decomposed components in the 1ML sample.

	$\widehat{{A}}~(\sigma _{A})$	$\widehat{{E}}~(\sigma _{E})$ (eV)	$\widehat{{w}}~(\sigma _{w})$ (eV)	$\widehat{{\eta }}~(\sigma _{\eta })$	$\widehat{{h}}~(\sigma _{h})$
SiC	72.4 (1.4)	283.7905 (0.0019)	0.7070 (0.0048)	0.100 (0.031)	2.58 (0.13)
Gr	23.9 (2.1)	284.766 (0.024)	0.943 (0.041)	0.44 (0.17)	
S1	24.8 (2.6)	285.050 (0.080)	0.91 (0.19)	0.20 (0.22)	
S2	48.3 (2.5)	285.691 (0.028)	0.953 (0.031)	0.461 (0.086)	

Table 2 summarizes the results of Bayesian spectroscopy for the 1ML sample, where ${\hat{\theta }}$ and $\sigma _{\theta }$ are the MAP estimates and the standard deviations of $P(\theta |\varvec{D}, \hat{b})$ as measures of the precision of the estimation.

**Figure 3:**
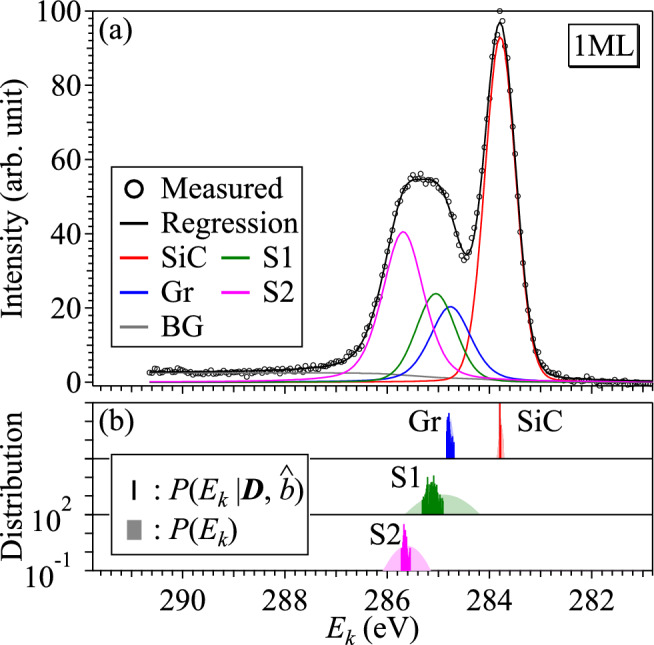
(**a**) Measured XPS spectrum, a regression spectrum and the decomposed spectral components for the 1ML sample. (**b**) Prior and posterior probability of the binding energy for each component.

The colored and black curves in Fig. 3a are the spectral components decomposed and a regression spectrum by Eq. (6), and Bayesian spectroscopy can successfully decompose the XPS spectrum into four components and the background signal with high reproducibility. The root mean square deviation (RMSD) of the regression spectrum is 0.83 in the intensity scale of the XPS signal, and it is consistent with the noise intensity ${\hat{\sigma }}_{\mathrm{noise}}=0.86$ ($=\hat{b}^{-1/2}$) estimated by the optimal quasi-inverse temperature $\hat{b}=1.36$ in Eq. (3).

Figure 3b shows the prior and posterior probability distributions of the binding energy $E_{k}$, in which the light and dark colors mean the prior and posterior ones, and the ordinate is on a logarithmic scale. Although the posterior probability distribution of Gr is as broad as its prior probability, the posterior probability distributions become narrower in the components SiC, S1, and S2, indicating that $E_{k}$ can be estimated with high precision. The probability distributions for S1 and S2 are particularly noteworthy in Fig. 3b. Although the prior probability distributions of S1 and S2 shown in light green and light magenta have overlapping hems, the posterior probability distributions shown in dark green and dark magenta are unimodal and noticeably narrower with no overlap, allowing us to decompose the hump structure into two distinguishable components with statistical assurance.

**Table 3:** MAP estimates and standard deviations of decomposed components in the 2ML sample. ^a^ we assume $w_{{\mathrm{S}}1} = w_{{\mathrm{S}}2}$ in the 2ML sample.

	$\widehat{{A}}~(\sigma _{A})$	$\widehat{{E}}~(\sigma _{E})$ (eV)	$\widehat{{w}}~(\sigma _{w})$ (eV)	$\widehat{{\eta }}~(\sigma _{\eta })$	$\widehat{{h}}~(\sigma _{h})$
SiC	36.2 (1.3)	283.7832 (0.0056)	0.688 (0.012)	0.021 (0.027)	3.79 (0.13)
Gr	66.3 (1.6)	284.7601 (0.0016)	0.6540 (0.0064)	0.552 (0.083)	
S1	15.4 (1.2)	285.089 (0.064)	1.199 (0.056)	0.08 (0.10)	
S2	33.4 (1.1)	285.515 (0.035)	N/A^a^	0.49 (0.17)	

Table 3 also summarizes the results of Bayesian spectroscopy in the 2ML sample. The binding energy of S2 in the 2ML sample is parameterized by the difference $\Delta {}E$ from the binding energy of S1. The MAP estimate of $\Delta {}E$ is 0.426 eV and the standard deviation of its posterior probability distribution is 0.056 eV ($<0.07~\text {eV}$). The binding energy of S2 shown in Table 3 is the result obtained by sampling of $E_{{\text {S}}1}+\Delta {}E$.

**Figure 4:**
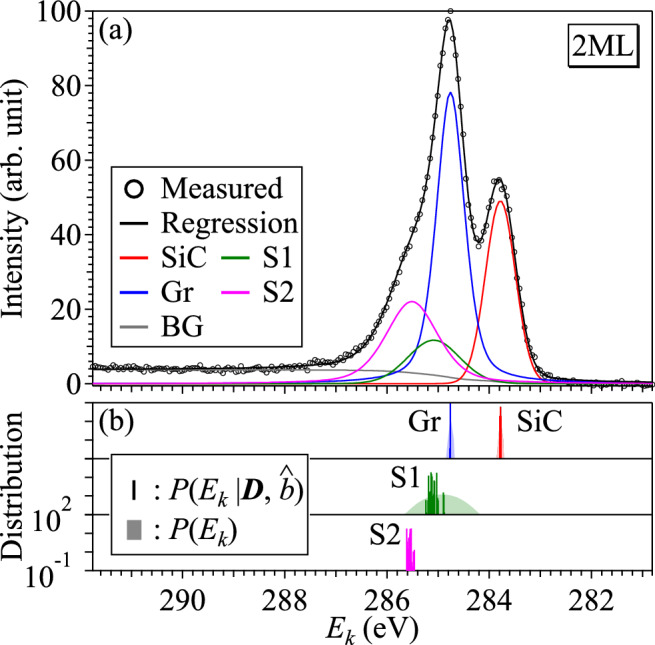
(**a**) Measured XPS spectrum, a regression spectrum and the decomposed spectral components for the 1ML sample. (**b**) Prior and posterior probability of the binding energy for each component.

Figure 4a also shows the results of Bayesian spectroscopy in the 2ML sample, and the regression spectrum indicated by a black curve can reproduce the measured one well, and the shoulder structure is explained by the components S1 and S2. In contrast to the prior probabilities of the binding energies of SiC, Gr, and S1 shown in light colors in Fig. 4b, their posterior probability distributions shown in dark colors are sharp and can be estimated with high precision. Although the prior probability distribution of $E_{{\text {S}}2}$ is not shown in Fig. 4b, because $E_{{\text {S}}2}$ is parameterized by $E_{{\text {S}}1}$ and $\Delta {}E$ and if these two parameters are independent of each other, the standard deviation of its prior probability is 0.338 eV ($=\sqrt{0.33^{2}+0.07^{2}}$) and is comparable to that (0.33 eV) of the prior probability of $E_{{\text {S}}1}$. As shown in dark magenta in Fig. 4b, the standard deviation of the posterior probability distribution for $E_{{\text {S}}2}$ is sufficiently smaller than its standard deviation. Such highly precise estimation can be achieved by incorporating the findings of the 1ML analysis as the energy difference between S1 and S2.

### Qfs-2ML sample

To estimate whether both the S1 and S2 components are included in the shoulder structure in Fig. 1c for the qfs-2ML sample, we prepare models with and without the S2 component and perform model selection. The BFEs of the model with and without S2 are 357.9 and 364.4, respectively. As a result, according to Eq. (3), Bayesian spectroscopy chooses the former model that includes the S2 component. The difference in these BFEs is Bayesian statistically apparent, and Bayesian spectroscopy argues that S2 is required for the XPS spectrum of the qfs-2ML sample even after accounting for the superimposed noise intensity ${\hat{\sigma }}_{\mathrm{noise}}=1.15$ ($\hat{b}=0.754$).

**Table 4:** MAP estimates and standard deviations of decomposed components in the qfs-2ML sample using the model with S2.

	$\widehat{{A}}~(\sigma _{A})$	$\widehat{{E}}~(\sigma _{E})$ (eV)	$\widehat{{w}}~(\sigma _{w})$ (eV)	$\widehat{{\eta }}~(\sigma _{\eta })$	$\widehat{{h}}~(\sigma _{h})$
SiC	56.03 (0.51)	283.0144 (0.0024)	0.8730 (0.0051)	0.002 (0.026)	3.159 (0.071)
Gr	51.50 (0.17)	284.0183 (0.0020)	0.5176 (0.0045)	0.186 (0.038)	
S1	12.2 (4.4)	284.541 (0.097)	1.31 (0.15)	0.16 (0.11)	
S2	0.5 (4.3)	284.97 (0.15)	1.21 (0.30)	0.63 (0.15)	

Table 4 summarizes the results of Bayesian spectroscopy in the qfs-2ML sample.

**Figure 5:**
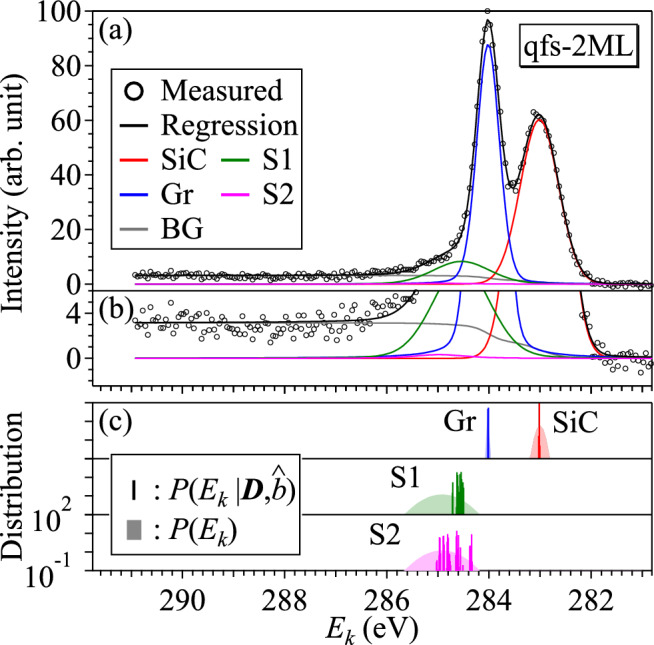
(**a**) Measured XPS spectrum, a regression spectrum and the decomposed spectral components for the qfs-2ML sample. (**b**) Enlarged ordinate scale of (**a**) to show the weak S2 component. (**c**) Prior and posterior probability of the binding energy for each component.

The color and black curves in Fig. 5a are the decomposed spectral components and a regression spectrum for the qfs-2ML sample, respectively. The black curve shows high reproducibility with the measured one, and the estimated $\hat{b}$ is consistent with the RMSD of the regression.

Figure 5b shows on an enlarged ordinate scale. Although we can confirm the weak S2 component shown in magenta, it is found that the contribution of S2 is quite small. The posterior probability distribution of $E_{{\text {S}}2}$ is wider than that of $E_{{\text {S}}1}$ in Fig. 5c. Bayesian spectroscopy searches for a globally optimal solution that reproduces the entire data using a model function, and it is possible to extract even components whose peak intensity is lower than the noise intensity^35^, such as the S2 component in this case.

**Figure 6:**
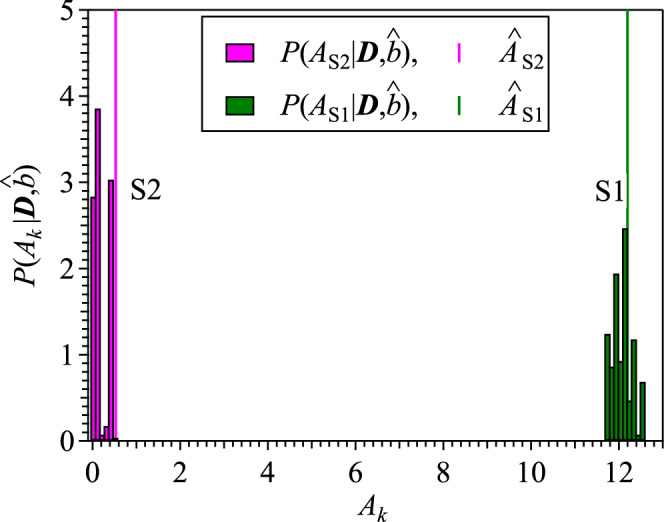
Posterior probability distributions of the integrated intensity for S1 and S2 components in the qfs-2ML sample. MAP estimates $\hat{A}_{{\text {S}}1}$ and $\hat{A}_{{\text {S}}2}$ are indicated by vertical lines.

However, we have to evaluate the integrated intensity $A_{k}$ to discuss the presence of the buffer layer S2, and Figure 6 shows the posterior probability distributions of $A_{{\text {S}}1}$ and $A_{{\text {S}}2}$ for components S1 and S2, in which the MAP estimates $\hat{A}_{{\text {S}}1}$ and $\hat{A}_{{\text {S}}2}$ are indicated by vertical lines. Bayesian spectroscopy, in fact, selects the model that includes the S2 component and gives a non-zero MAP estimate ($\hat{A}_{{\text {S}}2} = 0.5$) as shown in Table 4. However, the posterior probability of $A_{{\text {S}}2}$ is distributed near zero within the non-negative value constraint, and its standard deviation is as large as $\sigma _{A_{{\text {S}}2}} = 4.3$ ($>\hat{A}_{{\text {S}}2}$). The results of this analysis indicate that the annealing procedure in air terminates the dangling bonds of Si atoms under the buffer layer with oxygen atoms, causing the S2 component to disappear. It also means that there are almost no dangling bonds between the SiC substrate and graphene in the qfs-2ML sample. It is reported that an ideal oxidization proceedure can forms Si$_2$O$_5$ adlayer without dangling bond on Si-face of SiC substrate^36^. All of the dangling bonds and the covalent bonding between the buffer layer and SiC substrate would be terminated if our annealing proceedure fully oxidize the SiC substrate. $\hat{A}_{{\text {S}}2}$ in Table 4 is approximately 1/24th of $\hat{A}_{{\text {S}}1}$ for the S1 component, which is considered to be the S1 component that remained slightly after annealing.

## Discussion

According to the previous study^5^, the S1 component results from the C atoms bound to Si of SiC surface and C atoms in the buffer layer, which is approximately one third of the total C atoms in the buffer layer, and the S2 component is the result of the remaining C atoms in the buffer layer. In a previous study^23^, 0.31 has been reported for the integrated intensity of the S1 component relative to the sum of the S1 and S2 components in both 1ML and 2ML samples. We obtain results consistent with these previous studies^5,23^. We evaluate the posterior probability distributions of $\varLambda :=A_{{\text {S}}1}/(A_{{\text {S}}1}+A_{{\text {S}}2})$ from the sampling histories of $A_{{\text {S}}1}$ and $A_{{\text {S}}2}$, and obtain MAP estimates of 0.339 for the 1ML sample and 0.315 for the 2ML sample, respectively. The standard deviations of their posterior probability distributions are 0.032 and 0.020, respectively, and the values of previous studies are included within these ranges.

**Figure 7:**
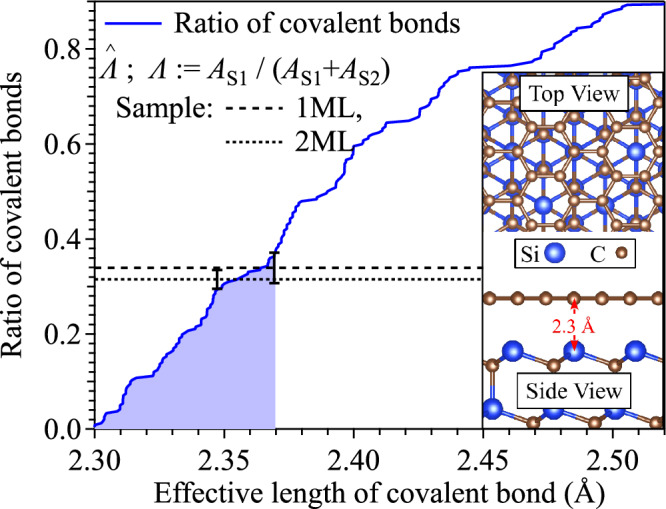
The number ratio of C covalently bonded to Si as a function of the effective length of its covalent bond. The horizontal lines and their error bars are the MAP estimates ${\hat{\varLambda }}$ and the standard deviations of the posterior probability distributions of $\varLambda$. The structural model in the inset was drawn using VESTA^37^ software.

We can estimate the effective length of the covalent bond, which gives S1, between C and Si based on this intensity ratio $\varLambda$, assuming that the intensities of the XPS signal of the components S1 and S2 are equivalent and using a structural model. A portion of the structural model at the interface of the graphene and SiC(0001) substrate is shown in the inset of Fig. 7. We consider a rectangular area of $34.045\times 58.971~{\text{\AA }}^2$ on the SiC surface tiled with $(\sqrt{3}\times \sqrt{3})~R30^{\circ }$ unit, in which the unit cell of graphene (brown honeycomb) rotates with $30^{\circ }$, and 242 Si atoms and 768 C atoms are interfaced in this rectangular area. The distance between the SiC substrate and graphene is 2.3 Å^38^. When the effective length of the covalent bond formation is extended to increase the number of covalent bonds with Si within the effective length, the ratio of the covalent bonds to all C atoms in the buffer layer increases as indicated by the blue curve in Fig. 7. The horizontal lines and their error bars are the MAP estimates ${\hat{\varLambda }}$ for the intensity ratio of the S1 components to the sum of S1 and S2 components and the standard deviations of the posterior probability distributions of $\varLambda$. Taking into account the accuracy of the estimation of ${\hat{\varLambda }}$, the measured S1 intensity ratios ${\hat{\varLambda }}$ can be understood by covalently bonding to Si at distances less than 2.37 Å, shown in a light blue area in Fig. 7. This is also consistent with a previous study^39^.

The primary advantage of Bayesian spectroscopy is that it provides estimates through statistical sampling in the parameter space. However, when multiple spectral components are expected to be contained in the tail part of a strong spectral structure and in a hump structure, as analyzed in this paper, the results of simple statistical sampling are deceptive. Our proposed method illustrated in Fig. 2 solves this problem and makes it possible to estimate the material-specific parameters with high accuracy. Consequently, the standard deviations of the posterior probability distribution for the binding energy, shown in Tables 2, 3 and 4 as measures of the accuracy of the estimation of the MAP estimates, are on the order of 100 meV even for the S2 component of the most severe case of the qfs-2ML sample, and are less than several 10 meV for the others, making an extremely accurate estimation of the binding energies.

## Conclusion

Using Bayesian spectroscopy, we have investigated the XPS spectra of graphene samples in the C 1s level. To perform the highly precise spectral decomposition of the XPS, we first performed an exhaustive search to explore the parameter space and then performed spectral decomposition by Bayesian spectroscopy using designed prior probabilities that incorporate the information based on physical properties and the insights gained from the posterior probability distributions evaluated in the exhaustive search. We have succeeded in decomposing the XPS spectra of the 1ML, 2ML, and qfs-2ML samples to the components: graphene, SiC, and the buffer layer atom(s) and estimation of the binding energies has been achieved with high precision of the order of meV. From their binding energies, we estimated the existence ratio of the buffer layers S1 for S2 with its standard deviation, which is consistent with previous studies. We performed model selection to determine the number of components in the XPS spectrum of the qfs-2ML sample. The four-peak model is selected, however, the contribution of S2 is quite small and this is probably considered due to the heterogeneity of the qfs-2ML graphene sample. These results demonstrate that the appropriate design of the prior probability distributions based on the information of physical properties and the insights gained from the exhaustive search is effective to perform the spectral decomposition with high precision.

## Data Availability

The data that support the findings of this study are available from the corresponding author upon reasonable request.
